# Supplementary material for: WeNet-RF: An Automatic Classification Model for Financial Reimbursement Budget Items
Source: PLoS One. 2025 Apr 24;20(4):e0321056. doi: 10.1371/journal.pone.0321056 (PMC12021194; doi:10.1371/journal.pone.0321056)
Supplement: Attachment — Submitted filename: Response to comments from Reviewer 2.docx [file pone.0321056.s002.docx]

**Manuscript Number PONE-D-24-45687**

**Automatic Classification Model for Financial Reimbursement Budget Items Based on WeNet-Random Forest**

Dear Reviewer,

Thank you very much for your detailed and constructive feedback. We have carefully reviewed your comments and made the necessary revisions to address each concern. At the same time, we have changed the title of the paper to: "WeNet-RF: An Automatic Classification Model for Financial Reimbursement Budget Items". Below are our detailed responses:

Question 1: The manuscript describes a technical solution to a real-world problem. However, the technical definitions of model controls and parameter tuning were not shown. Additionally, the model training process and the number of epochs were not described. The simulation experiments and the number of experiments conducted were also not described. Only the mean values of the experiments were shown.

*Response 1: We have added detailed descriptions of the hyperparameter tuning process in Section 2.6 (Random Forest Model). Specifically, we have included information on the tree depth and other critical parameters used in the Random Forest model (Lines 292-294). In Section 2.7 (Cross-Validation Assessment), we have clarified the number of simulation experiments conducted and the statistical methods used to analyze the results (Lines 317-318). These additions provide a comprehensive understanding of the model's training and evaluation processes.*

Question 2: I cannot ascertain if the right statistical procedures were followed and rigorously carried out. Though literary descriptions were presented, there were no technical details to support and validate those descriptions in terms of model equations and simulation experiments.

*Response 2: We understand the importance of rigorous statistical validation. To address this, we have provided detailed descriptions of the statistical methods used in the study, including the equations and simulation experiments. This information is now included in the relevant sections of the manuscript, ensuring that readers can fully understand and reproduce our results.*

Question 3: The sample data used for the research were described, stating how they were acquired and the dataset size. However, the authors require access to research data through an email request due to the highly sensitive nature of financial data.

*Response 3: We acknowledge the journal's policy on data availability. While we understand the importance of making data publicly available, the financial data used in this study are highly sensitive and cannot be shared publicly. We have added a clear statement in the "Data Availability Statement" section, indicating that the dataset can be obtained by contacting the teachers of the school's financial department via email (Name: Zeng Tingting; Contact number: +86 871-68305391; Email address: 1908584074@qq.com). We are committed to facilitating access to these data for researchers who meet the necessary criteria.*

Question 4: The manuscript was well presented in a logical and intelligent manner, clearly written in standard English. However, the reporting is required to be done in the past tense since the research has been completed. Some acronyms that were used at first instance (i.e., MODIS, ASR, etc.) were not expanded in full.

*Response 4: We have carefully reviewed the manuscript and revised the language to ensure that all descriptions are in the past tense, reflecting the completed nature of the research. Additionally, we have expanded all acronyms at their first appearance to avoid confusion for readers. These changes enhance the clarity and consistency of the manuscript.*

We hope that these revisions address all of your concerns. We appreciate your valuable feedback and look forward to your positive response.

Thank you once again for your time and consideration.

Best regards,

Peichun Suo
